# ISBER and the Biobanking and Cohort Network (BCNet): A Strengthened Partnership

**DOI:** 10.1089/bio.2018.29043.mkh

**Published:** 2018-10-12

**Authors:** Marianne Krall Henderson, Zisis Kozlakidis

**Affiliations:** ^1^ Center for Global Health, National Cancer Institute, National Institutes of Health, DHHS, Bethesda, Maryland.; ^2^ International Agency for Research on Cancer (IARC), World Health Organizaton (WHO), Lyon, France.

The Biobanking and Cohort Network (BCNet) was established by the International Agency for Research on Cancer (IARC) in 2013, as an opportunity for low- and middle-income countries (LMICs) to work together in a coordinated manner to jointly address many of the challenges in biobanking infrastructure, the acquisition and maintenance of high-quality samples and data, and governance and regulatory frameworks to guide in the sharing and reuse of resources for research. The BCNet currently has 34 LMIC member organizations from across the world and 13 partner organizations that have biobanking in their core activities. The BCNet initiative arose from the realization that despite improvements in developed countries, population cohorts and biobanking facilities are either underdeveloped or nonexistent in LMICs.^1^ In this context and in line with IARC's mission to contribute to worldwide cancer research, BCNet was set up as an opportunity for LMICs to work together in a coordinated and effective manner and jointly address the many challenges in biobanking infrastructure, including ethical, legal, and social issues.^2,3^ In addition, BCNet facilitates the sharing of resources (e.g., expertise and protocols) and the development of joint projects, strengthening the competitiveness of LMIC biobanks in applying for international funding. IARC's Dr. Maimuna Mendy has expertly coordinated the BCNet with the help of a steering committee team focused on education and training, ethics and legal issues, and informatics (http://bcnet.iarc.fr). The BCNet steering committee has organized several training initiatives and resources; it has focused on the informatics necessary to support LMIC biobanking, in partnership with B3Africa; and has examined ethics and legal issues within and across LMIC biobanking, including sample and data sharing.^4^

The International Society for Biological and Environmental Repositories (ISBER) is one of the founding partner organizations of the BCNet and has taken a key role in the education and training portion of its outreach activities. ISBER entered into a partnership agreement with IARC-BCNet in 2015 to allow very low-cost access to the ISBER educational platform and the ISBER online biobanking training modules, Introduction to Biobanking, developed by the Canadian Tissue Resource Network. The purpose of the course is to give the viewer a general overview of the key issues in establishing, maintaining, and accessing a biobank. The education program includes nine online modules designed to provide “how-to” knowledge for researchers and biobankers. This course is currently available in the English and French languages.

Now ISBER, in line with its mission to strengthen international collaboration^5^ and based on further recent evidence,^6^ has entered into a stronger partnership agreement with IARC BCNet. ISBER has made a firm commitment over the past few years to lower costs for resource-constrained biobankers and organizations, in addition to the partnership with IARC-BCNet. The new agreement between the two organizations welcomes all registered LMIC organizations that are members of BCNet into ISBER in a complimentary membership capacity. This new status provides BCNet members with full access to ISBER tools, including the ISBER forum discussion board, participation in working groups and committees, the ISBER best practices, science policy resources, and recorded conference sessions and webinars.

In addition, BCNet members will be able to communicate with the ISBER membership through the provision of complimentary news features and the creation of a joint working group or task force for developing further educational material that would be LMICs specific and freely available to the members of both organizations. ISBER looks forward to this newly strengthened relationship to support our strategic commitment to education and harmonization of best practices in biobanking across the world, in all resource settings.
